# Signal Transduction of Fertilization in Frog Eggs and Anti-Apoptotic Mechanism in Human Cancer Cells: Common and Specific Functions of Membrane Microdomains

**DOI:** 10.2174/1874091X00802010049

**Published:** 2008-04-29

**Authors:** Ken-Ichi Sato

**Affiliations:** Address correspondence to these authors at the Laboratory of Cell and Developmental Biology, Department of Biotechnology, Faculty of Engineering, Kyoto Sangyo University, Kamigamo-Motoyama, Kyoto 603-8555, Japan

## Abstract

Membrane microdomains or lipid/membrane rafts are distinct areas on the plasma membranes, where a specific subset of lipids (e.g. cholesterol, sphingolipids) and proteins (e.g. glycosylphosphatidylinositol-anchored proteins, growth factor receptor/kinases) are getting together and functioning for several aspects of cellular functions. Our recent investigation has revealed that fertilization of African clawed frog, *Xenopus laevis*, requires cholesterol-dependent nature of egg membrane microdomains. Moreover, fertilization of *Xenopus* eggs involves proteolytic cleavage of the extracellular part and subsequent phosphorylation of a cytoplasmic tyrosine residue of uroplakin III, an egg membrane microdomain-associated protein. Protease activity toward uroplakin III seems to be derived from fertilizing sperm, while phosphorylation of uroplakin III seems to be catalyzed by the egg tyrosine kinase Src, whose activation is required for cytoplasmic rearrangement of fertilized eggs; so-called ‘egg activation’. Therefore, it is assumed that uroplakin III serves an integral part of signal transduction in fertilization of *Xenopus*. Our more recent study on human cancer cells has revealed that a similar but distinct scheme of signal transduction operates in anti-apoptotic growth of cells. Namely, in human bladder carcinoma cells, cooperation of uroplakin III and Src, both of which localize to the membrane microdomains, allows cells to escape from apoptotic cell death and proliferate under culture conditions deprived of serum. In this review, I briefly introduce about biology of fertilization and cancer, and then present and discuss our experimental data on general importance and specific features of membrane microdomains in *Xenopus* fertilization and anti-apoptosis in human bladder carcinoma cells.

## SIGNAL TRANSDUCTION OF FERTILIZATION

### Overview of Fertilization Research

Fertilization is the union of two gamete cells; egg and sperm that combines two paternally prepared genetic materials and that gives rise to a newborn with a distinct genetic background to their parents [1-7]. Although recent advances in biotechnology (i.e. somatic cell nuclear transfer) has enabled us to create cloned animals in some species including mammals [7], whose genetic materials are practically as same as their donor animals, there has been no exception in that fertilization is the only mean for natural offspring in sexual reproduction system. Therefore, to understand molecular and cellular mechanism of fertilization has been a long-thought theme in biology and medicine, by which we may establish more effective strategies for medical and pharmaceutical treatments, for agricultural and industrial productions, for taking care of environmental issues, and most of all, for understanding of ourselves, human being. Fertilization, by its terminology, cover several successive aspects of reproduction; including gametogenesis (oogenesis/spermatogenesis and acquisition of ability to be fertilized), gamete interaction with reproductive tract (especially in mammals) and with each other, gamete fusion, activation of egg/oocytes, and establishment and initiation of zygotic development (including implantation in mammals). Practically, however, the research field of fertilization covers more deeply inside from sperm-egg interaction through initiation of development than other processes. In this view, the following three questions have been asked as general and major questions in the fertilization field. How does sperm (or egg) recognize and interact with egg (or sperm)? [1-4] How do sperm and eggs fuse with each other? [8,9] How does fertilized egg get activated to initiate embryonic development? [10-13]

### How do Sperm and Egg Interact and Fuse with Each Other?

**Fig. (1):**
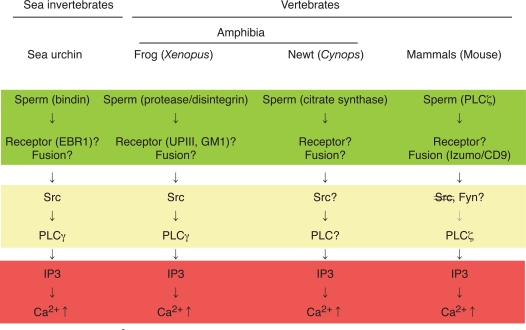
Signal transduction leading to Ca^2+^transient at fertilization in sea urchin, Xenopus laevis (frog), Cynops pyrrhogaster (newt), and mouse. Shown schematically is sequence of events at fertilization in four model organisms (sea urchin as representative of sea invertebrates, frog and newt as representative of amphibian vertebrates, and mouse as representative of mammalian vertebrates). In all species, IP3-dependent mechanism is a dominant component of intracellular Ca^2+^ release (as highlighted in red), which is necessary for successful egg activation by sperm. In most species, molecular detail of sperm-egg membrane interaction and fusion is unknown and may be varied between species (as highlighted in green). Special note that genetic approach has identified sperm Izumo and egg CD9 as essential components for sperm-egg fusion. Egg cytoplasmic events connecting sperm-egg interaction/fusion and IP3-dependent Ca^2+^ release are semi-conserved among species and involve tyrosine kinase-dependent or-independent actions of PLCs (as highlighted in yellow)

A number of studies have been conducted to answer aforementioned questions by using several model organisms. Not only vertebrates like mammals, birds, reptiles, amphibian, and fishes, but also invertebrates such as insects, nematodes, and sea creatures have been employed. Fig. (1) depicts current state of our knowledge on a sequence of events associated with fertilization, in other words, signal transduction of fertilization, by highlighting four organisms analyzed extensively; sea urchin, frog, newt, and mouse. At present, sperm-egg interaction at the plasma membrane level is most uncertain subject. Biochemical and immunochemical studies in the mouse have identified some candidates for sperm-egg membrane interaction; for instance, a disintegrin and metalloproteinase or ADAMs in sperm and integrins in eggs [14,15]. However, genetic deletion of these molecules by gene knockout could not eliminate the ability of eggs or sperm to undergo membrane interaction, indicating that these molecules are not essential for this process [16-18]. Until now, no gene knockout experiments have succeeded in obtaining phenotype that sperm-egg interaction is impaired. On the other hand, the same experimental approach has identified two gene products, egg CD9 [19-21] and sperm Izumo [8,9,22], as essential components for sperm-egg membrane fusion in mouse. CD9 is a member of tetraspanin molecules that contain four transmembrane domains as well as two extracellular loops and two cytoplasmic sequences. It is also known that CD9 constitutes a protein complex with certain kinds of integrin, a cell adhesion protein, and serves as membrane organizer [23-27]. Recent reports have demonstrated that fragments of egg membranes containing CD9 are transferred to sperm surface, by which sperm may acquire the ability to fertilize egg [28]. It should also be noted that sperm surface also express functional integrin complexes that seem to be important for sperm-egg interaction and fusion [29]. Izumo processes an immunoglobulin-like domain in its extracellular sequence [22], which has been implicated in molecular interaction in several other immunoglobulin-like domain-containing proteins. Both CD9 and Izumo proteins, however, do not possess intrinsic activity for membrane fusion like SNARE complex. Therefore, it seems that these two proteins act to orchestrates structural assembly and/or function of membrane-associated molecules directly involved in fusion process.

### Membrane Events Leading to Egg Activation at Fertilization

Gene knockout approach is not yet available in other species presented in Fig. (1). Instead, much larger number as well as size (or volume) of eggs that can be obtained from one animal has enabled researchers to conduct more detailed biochemical and cell biological experiments. It is noteworthy that adult, female mouse would give us ~10 eggs with a diameter of ~200 µm per ovulation, while adult, female frog would give us ~1000 eggs with a diameter of ~1300 µm per ovulation. In sea urchin, sperm post-acrosomal protein bindin [30] and egg EBR1 [31,32] have been identified and characterized as components for species-specific interaction of gametes at the egg vitelline membrane level, although its relationship to gamete fusion and egg activation is unclear. In Xenopus, a sperm protein containing disintegrin sequence, named xMDC16 [33,34], has been suggested to be a component to interact with and activate egg. Synthetic peptides containing disintegrin motifs, such as Lys-Thr-Cys [34], which is present in the xMDC molecule, and Arg-Gly-Asp [35], which has not yet been identified in any of Xenopus sperm proteins, has been shown to activate Xenopus eggs, suggesting the presence of functional integrin(s) on egg surface. Several types of integrin (heterodimer of α and β subunits) have been identified in Xenopus [36]; however, their involvement in sperm-egg interaction and egg activation has not yet been demonstrated. Another candidate mechanism for sperm-egg interaction in Xenopus involves proteolysis [37]. External application of protease such as cathepsin B promotes egg activation in Xenopus, suggesting that sperm-derived cathepsin B-like protease, if present, is involved in gamete interaction and egg activation. In support with this, specific inhibitors for cathepsin B inhibits not only cathepsin B-induced egg activation but also sperm-induced egg activation. As a candidate for protease substrate on the egg surface, a single transmembrane protein uroplakin III has been identified [38, for detail see below]. One question arises as to whether such protease-substrate interaction is sufficient for cellular interaction done by egg and sperm. Association of uroplakin III with egg membrane microdomains and its possible functions in sperm-egg interaction and egg activation will be discussed later. Molecular detail of sperm-egg fusion in Xenopus is, as in case of sea urchin, not well understood. The fact that CD9, an egg molecule that is essential for sperm-egg fusion in the mouse, is also present in Xenopus eggs [39] would allow researchers to evaluate the function of this molecule in the near future. In summary, mechanisms of sperm-egg interaction and fusion seems to be quite different among model organisms and its understanding at molecular level awaits further investigation.

### Mechanism of Ca^2+^ Transient at Fertilization 

The term “egg activation” means a sum of biological events that culminates in activation of embryonic development. It includes resumption of the egg’s cell cycle (from the metaphase of meiosis II in most mammals and amphibians), block to polyspermy, fusion of female and male nuclei, and synthesis of zygotic genome DNA [2,4,6,40]. Cytoplasmic events leading to egg activation have been analyzed extensively in many animal species, and it is well established that transient rise of intracellular Ca^2+^ concentration, in short Ca^2+^ transient, plays a universal role in executing egg activation [2,4-6,41]. In other words, egg activation is dependent on the occurrence of Ca^2+^ transient. In support of this, artificial elevation of intracellular Ca^2+^ concentration can reproduce essentially all events of egg activation. Upstream events of Ca^2+^ transient are also conserved among species; at least in four organisms shown in Fig. (**1**), phospholipase C-dependent production of inositol 1,4,5-trisphosphate (IP3) act to trigger Ca^2+^ transient, which will initiate and propagate from the endoplasmic reticulum (Fig. **1**). Thus, it is interesting to note that, compared to diverse nature of membrane events of fertilization, cytoplasmic mechanism of fertilization are highly conserved among species. Phospholipase C (PLC) is a family of enzymes that comprises of six subspecies: β, δ, γ,ε, η, and ζ  [42,43]. Among them are PLCγ and PLCζ, whose involvement in sperm-induced IP3 production and Ca^2+^ transient has been demonstrated. In sea urchin and *Xenopus*, tyrosine phosphorylation of PLCγ, which is catalyzed by Src family tyrosine kinases, is reported to be important for egg activation [12,44]. Src-PLCγ pathway is well known to operate in several somatic cell systems such as T-and B-lymphocytes, where cell surface receptors activate this pathway in response to binding to antigenic ligands [45-47]. In analogy of this, it is believed that sea urchin and *Xenopus* may utilize sperm-egg membrane interaction to promote signal transduction pathways leading to egg activation, as discussed in detail later. On the other hand, in the mouse, sperm-derived PLCζ has been shown to be involved in initiating egg activation [13,48-51]. In this case, sperm-egg fusion, which allows sperm to deliver its constituents: not only genetic materials but also other components including PLCζ, seems to be a critical for initiating egg activation. Thus, upstream events of IP3 production and Ca^2+^ transient are semi-conservative among species, in which sperm-egg membrane interaction or fusion acts as an initiator of signal transduction pathways leading to activation of PLCs (Fig. **1**). It should be noted that, in the newt *Cynops pyrrhogaster*, another mechanism of Ca^2+^ transient has been show to operate. It involves sperm-egg membrane fusion and introduction of sperm-derived citrate synthase (Fig. **1**) [52]. Molecular mechanism by which citrate synthase induces Ca^2+^ transient (tyrosine kinase? PLC? IP3?) remains to be clarified.

## MEMBRANE MICRODOMAINS AND FERTILIZATION SIGNALING IN XENOPUS

### Overview of Membrane Microdomains or “Rafts”

**Fig. (2):**
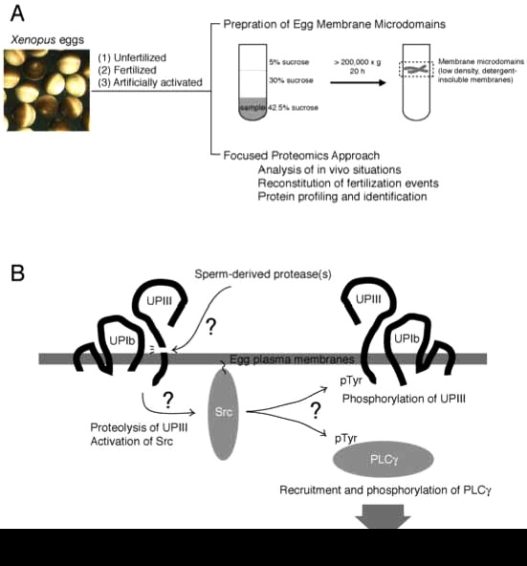
Study of fertilization signaling focusing on structure and function of egg plasma membrane microdomains. (A) Schematic drawing of preparation of membrane microdomains from Xenopus eggs, which are unfertilized, fertilized, or artificially activated (parthenogenetically activated), and its utility in functional analysis. (B) Schematic diagram showing a series of events that operates during early phase of fertili-zation of Xenopus eggs. Sperm-induced egg activation may involve proteolytic cleavage of the egg membrane microdomain-associated uro-plakin III, catalyzed by sperm-derived protease. Uroplakin III is in association with uroplakin Ib, a tetraspanin molecule that supports the localization of uroplakin III to membrane microdomains. The uroplakin III-uroplakin Ib complex is involved in negative regulation of cyto-plasmic tyrosine kinase Src, and proteolytic cleavage of uroplakin III may lead to up-regulation of Src by unknown mechanism. Activated Src phosphorylates and activates PLC,γ which in turn promotes IP3 production and Ca^2+^transient.Src may also phosphorylate intact uro-plakin III, although its physiological importance has not yet been demonstrated

The term “membrane microdomains” or “membrane/lipid rafts” denotes a distinct area or micro-segment on the plasma membranes, where a specific subset of proteins and membrane lipids are getting together and working for certain cellular functions [53-59]. It is sometimes called as “cavelolae microdomains” or more simply “caveolae”, when it involves invaginated membrane structures containing caveolin, a membrane-spanning protein [56]. With experimental basis, membrane microdomains are also called as “detergent-insoluble membrane (DIM)” or “detergent-resistant membranes (DRM)” [53,57,58]. Such detergent-insoluble or resistant nature of membrane microdomains is due to an enrichment of cholesterol and saturated fatty acids that constitute liquid-ordered membranes. Conventional preparation of membrane microdomains involves extraction of cell samples with Triton-X-100 (or other non-ionic detergents)-containing buffer at low temperature, followed by fractionation of the extracts with discontinuous sucrose gradient ultracentrifugation (Fig. **2a**). The resulting fractions containing low-density, detergent-insoluble materials are collected as membrane microdomains. For further details of technical issues and merits of focused approach on egg membrane microdomains in fertilization study, please refer to other literatures [60,61].

### Characterization of Membrane Microdomains of Xenopus Eggs

As discussed earlier, quality amounts of eggs in *Xenopus* system has allowed us to perform biochemical characterization of signal transduction pathways leading to egg activation at fertilization. Earlier works in our laboratory have demonstrated that sequential activation of tyrosine kinase Src and PLCγ are required for IP3 production and Ca^2+^ transient in fertilized *Xenopus* eggs [62-65]. Subsequent studies focusing on egg membrane microdomains have demonstrated the following: Conventional preparation of membrane microdomains (see above) has allowed us to obtain low-density, detergent-insoluble membrane fractions that are enriched in cholesterol, ganglioside GM1, and cytoplasmic tyrosine kinase Src [39], all of which are known membrane microdomain markers in many types of mammalian somatic cells. Fertilization promotes a rapid activation of membrane microdomain-associated Src, as judged by immunoblotting with use of phospho-tyrosine 416-specific antibody that recognizes activated Src molecules [39]. Fertilization also promotes a transient recruitment of PLCγ to membrane microdomains, by which it is tyrosine-phosphorylated and activated by Src [65,66]. Application of methyl-β-cyclodextrin, a cholesterol-binding substance that disrupts organization of membrane microdomains, abolishes the activation of Src and subsequent egg activation events in fertilized eggs. Importantly, localization of Src and PLCγ to membrane microdomains is distorted methyl-β-cyclodextrin-treated eggs [39,66]. These results focusing on *in vivo* situations of membrane microdomains (Fig. **2a**) suggest that at least the cytoplasmic face of egg membrane microdomains acts as a structural platform for Src-dependent egg activation signaling at fertilization. Our next experiments evaluating function of extracellular face of egg membrane microdomains have demonstrated the following:

### Focused Proteomics on Xenopus Egg Membrane Microdomains

Egg membrane microdomains isolated from unfertilized eggs have been shown to contain functional Src protein. Surprisingly, addition of sperm to this membrane microdomain preparation promotes activation of Src *in vitro* [66], indicating that sperm-interacting machinery, if any, may be functionally preserved in this preparation and that it could be useful for *in vitro* reconstitution of sperm-egg membrane interaction and subsequent signal transduction. With these assumptions, we have performed several biochemical and cell biological experiments examining the ability of isolated membrane microdomains to reproduce fertilization events. In combination with cell-free extracts prepared form unfertilized *Xenopus* eggs (so-called cytostatic factor-arrested or CSF extract), sperm-treated membrane microdomain could reproduce Ca^2+^ transient and subsequent egg activation processes that include resumption of meiotic cell cycle (as judged by the occurrence of morphological change of added sperm nuclei) and dephosphorylation of mitogen-activated protein kinase, an abundant protein serine/threonine kinase that regulates cell cycle of unfertilized eggs and early embryos in *Xenopus* [60,66]. Importantly, pharmacological inhibition or immunochemical depletion of Src or PLCγ shows an inhibitory effect on all of these reconstituted fertilization events. Thus, membrane microdomains prepared from *Xenopus* eggs have allowed us to perform to some extent *in vitro* reconstitution of signaling events at fertilization (Fig. **2a**).

### Uroplakin III, a Novel Component of Fertilization 

Focused proteomics approach on egg membrane microdomains has also gained a new insight for signal transduction at fertilization. Immunoblotting of egg membrane microdomain fractions with anti-phosphotyrosine antibody has demonstrated that a 30-kDa protein becomes tyrosine-phosphorylated in fertilized eggs (5 min after insemination). Mass spectrometry analysis has demonstrated that it is uroplakin III (UPIII) [67]. UPIII is a member of uroplakin family that consists of four members: UPIa and UPIb, both of which are tetraspanin molecules, and UPII and UPIII, both of which are single-transmembrane proteins [68,69]. UP family proteins are originally identified in bladder and kidney tissues of mammals. They have been implicated in organizing rigid nature of apical surface of urothelial membrane structures, which involves urothelial plaque containing UPIa/UPII and UPIb/UPIII heterogeneous protein complexes. In addition, UP family proteins have been implicated in urinary bladder carcinomas, and some renal and infectious diseases [69-73]. Before our discovery of UPIII in *Xenopus* eggs, however, no information has been available as to the function of UP family proteins in reproduction and early development. Molecular identification of UPIII as a predominantly tyrosine-phosphorylated protein in the membrane microdomains of fertilized eggs has led us to investigate its physiological importance of this protein. Several biochemical and molecular biological studies have demonstrated the following:

### Signal Transduction Involving Uroplakin III and Src in Xenopus Fertilization

UPIII localizes predominantly to the egg membrane microdomains before and after fertilization [67]. Exposure of the amino-terminal domain of UPIII on the egg surface has been confirmed by the fact that UPIII is effectively labeled by surface biotinylation of unfertilized *Xenopus* eggs [67]. The fact that fertilization promotes phosphorylation of UPIII on tyrosine-249, which locates in the cytoplasmic sequence of this protein (as determined by de novo sequencing with mass spectrometry), suggests that the egg cytoplasmic tyrosine kinase Src, which is also concentrated in membrane microdomains and activated upon fertilization (see above), catalyzes this phosphorylation. In support with this, a pharmacological Src inhibitor PP2 abolishes tyrosine phosphorylation of UPIII. Another supporting evidence is that co-expression of UPIII and Src in 293 human embryonic kidney cells results in effective tyrosine phosphorylation of UPIII [67,74]. Although physiological role of phosphorylation of the UPIII tyrosine-249 is currently unknown, it could be a specific binding site for certain phosphotyrosine-binding protein(s). One interesting candidate is PLCγ, which is recruited to membrane microdomains in fertilized eggs (see above), because this protein contains two Src homology 2 domains that can bind to certain phosphotyrosine-containing sequence. On the other hand, physiological importance of the extracellular domain of UPIII has been suggested by the fact that a specific antibody against the extracellular domain of UPIII shows an inhibitory effect on fertilization in a dose-dependent manner [67]. The results obtained with anti-UPIII antibody suggest that the extracellular domain of UPIII is required for sperm-egg interaction and/or fusion at fertilization.

### Uroplakin III is a Candidate Target for Sperm-Derived Protease

UPIII has been identified as a target of sperm-derived protease, which has an enzymatic property similar to cathepsin B and is essential for fertilization [38]. The antibody against the extracellular domain of UPIII seems to abolish the proteolytic action of sperm. A synthetic peptide that corresponds to a potential proteolytic site in UPIII (containing Gly-Arg-Arg sequence, a proteolytic motif for cathepsin B) has been shown to block proteolysis of UPIII by sperm protease *in vitro* and fertilization *in vivo* [38]. Although these results do not exclude the possibility that other cell surface molecules could also be an important substrate for sperm-derived protease, it is possible to surmise that UPIII is a part of protein complex for interacting with fertilizing sperm. In 293-cell expression system, UPIII requires UPIb for its proper localization to the membrane microdomains [72,74]. Co-expression of UPIb, UPIII and Src results in inactivation of Src, as judged by elimination of phosphorylation of tyrosine-416, as well as by decrease of tyrosine phosphorylation of other cellular proteins [74]. Thus, it is attractive to speculate that in the membrane microdomains of unfertilized eggs, UPIb/UPIII complex serves to negatively regulate Src and that upon fertilization, proteolytic cleavage of UPIII somehow releases the active form of Src inside the egg, thereby Src undergoes phosphorylation of important substrates such as UPIII and PLCγ (Fig. **2b**).

### Future Directions of the Research

Focused proteomics approach on membrane microdomains has also been conducted in sea urchin egg and sperm, and mammalian sperm [75-80]. In particular, sperm membrane microdomains are under extensive investigation with a focus on the roles of glycosphingolipids and other carbohydrates [79,80]. Further study, in combination with molecular biological approach, will be necessary to evaluate the physiological function of membrane microdomains in fertilization. In this sense, relationship of membrane microdomains and sperm-egg fusion system in the mouse (i.e. CD9/Izumo system) will be of quite interest.

## ANTI-APOPTOTIC GROWTH OF CANCER CELLS

### Overview of Biological Functions of Cancer Cells

Understanding of biological functions of cancer cells is of universal importance and prerequisite for establishing effective strategy for prevention, diagnosis, treatment and prognosis of cancer. Since our grasp of cancer as a result of aberrant action of several gene products that regulate normal cellular function, much effort has been made to characterize cellular functions peculiar to cancer cells and its molecular basis [81,82]. Apoptosis is a cellular mechanism that maintains a balance of various cellular functions through the active elimination of unnecessary cells from the living organism [83,84]. Its appropriate execution is important for proper animal development, namely early embryogenesis, later morphogenesis and body-shape-making. In adult, apoptosis is still important for balancing homeostasis and, as highlighted in this review, for preventing growth of aberrantly proliferating or malignantly transformed cells. All of normal cells, with an important exception in germ cells, will die according to their pre-programmed longevity, in other words, aging and senescence, in which proper arrangement of chromosomes will be distorted [85,86]. Even before the longevity, cells exposed to serious damages that include the exposure to ultraviolet, radioactivity, medicines etc. undergo cell death by apoptosis or necrosis, another style of cell death involving passive or unpredicted processes. In general, cancer cells arise from a summation of mutations occurred on some genes that are critically important for normal cell proliferation and/or cell death [81,82]. It means that cancer cells acquire not only the ability to proliferate aggressively but also the ability to escape from being killed by themselves (anti-apoptosis) or by other reasons (anti-longevity or anti-necrotic cell death). With an emphasis on anti-apoptotic mechanism of cancer cell function, our studies on human cancer cells have demonstrated the following:

### Analysis of Bladder Carcinoma Cells Focusing on its Resistance to Apoptosis

**Fig. (3):**
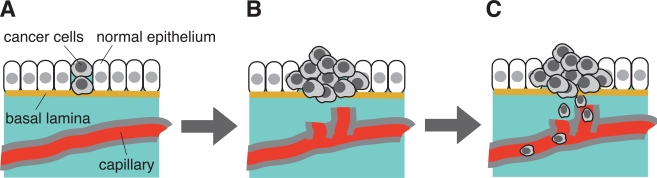
Initiation, promotion and progression of carcinogenesis: from benign tumor to malignant tumor. (A) Survival and growth under microenvironment where no supply of energy and nutrient is available. Serum starvation in cell culture may mimic such a microenvironment in vitro. (B) Anti-apoptotic mechanism of cancer cells. Cell become invasive and enter newly formed capillary (induction of angiogenesis). (C) Newly formed capillary supports growth and survival of tumor. Cells travel through bloodstream and undergo metastasis. This scheme is adapted from “ The Bi9ology of Cancer” by R.A. Weinberg (Garland Science, 2007).

There are several experimental conditions that promote apoptosis in cultured cancer cells. They include application of Fas ligand, a death inducer, or anti-Fas antibody, hypoxia, hydrogen peroxide, irradiation, and chemical inhibitors for metabolic enzymes or signaling pathways etc. [87-89]. Serum starvation serves as one of such pro-apoptotic cellular conditions examined to date. At the initial stage of carcinogenesis, cancer cells are not necessarily surrounded by capillary system. Under such microenvironment, however, cancer cells can proliferate effectively; thereby they are capable of inducing angiogenesis that will help cancer cells to populate more and to undergo invasion and metastasis (Fig. **3**). We have employed human bladder carcinoma cell line 5637 as a model cell system to analyze molecular mechanism of serum starvation-resistant growth of cancer cells [90-92]. Survival and growth of this cell line in serum-free conditions have been reported to involve the actions of ligands for epidermal growth factor receptor/kinase (EGFR/kinase) [93], suggesting that protein tyrosine phosphorylation plays a central part of survival signaling. In support of this, immunoblotting with anti-phosphotyrosine antibody has demonstrated that a number of proteins become tyrosine-phosphorylated in response to serum starvation [94]. Interestingly, time course of increase in tyrosine phosphorylation is quite slow; about eight hours are required for its plateau phase, indicating that up-regulation of EGFR/kinase ligands, if any, may need some hours to be promoted. Immunoprecipitation studies have demonstrated that 170-kDa EGFR/kinase is tyrosine-phosphorylated in serum-starved 5637 cells; however, it is different from a more prominently tyrosine-phosphorylated protein of 145 kDa. Mass spectrometric analysis has demonstrated that it is β-subunit of hepatocyte growth factor receptor/kinase (HGFR/kinase or c-Met). Although HGFR/kinase and its cognate high affinity ligand HGF/scatter factor (SF) have been originally identified in liver cells (hepatocytes), they are ubiquitously expressed in many kinds of cells and tissues, and implicated in a number of cellular functions (growth, differentiation, apoptosis etc.) [95,96]. Therefore, it is possible to think that, in addition to EGFR/kinase and its ligands, HGFR/kinase and HGF/SF are involved in the survival mechanism of 5637 cells under serum-free conditions.

### Mechanism of Anti-Apoptotic Function in Bladder Carcinoma Cells

Consistently with the previous findings by others that neutralizing antibodies to EGFR/kinase could abolish the growth of 5637 cells under serum-free conditions, a chemical inhibitor for EGFR/kinase (AG99) shows an inhibitory effect on growth of serum-starved 5637 cells. However, neither a potent inhibitor for HGFR/kinase (K252a [97]) nor a neutralizing substance for HGF/SF (Cu^2+^ ions [98]) showed such an inhibitory effect. Among other pharmacological kinase inhibitors challenged, Src-specific inhibitors (PP2 and SU6656 [99-101]) were most inhibitory to cell growth in serum-starved 5637 cells. Moreover, both EGFR/kinase inhibitors and Src inhibitors (but not HGFR/kinase inhibitor) were shown to block effectively tyrosine phosphorylation of the 145-kDa c-Met protein (hereafter p145^met^). Therefore, we conclude that tyrosine phosphorylation of p145^met^ is not due to autophosphorylation of the kinase, but rather due to trans-phosphorylation events catalyzed by EGFR/kinase and/or Src family kinases [94]. In some cancer cells (such as breast cancer), coordinate action of EGFR/kinase and Src has been shown to be required for their malignant phenotype [102-104]. So, our further study has focused on the roles of these two kinases in the molecular mechanism of survival in serum-starved 5637 cells.

### Tyrosine Phosphorylation-Dependent Mechanism of Anti-Apoptosis

Inhibition of cell growth in serum-starved 5637 cells in the presence of PP2 involves apoptosis, because concomitant increase of cell death and activation of caspase 3/7-like protease are observed [94]. Importantly, PP2 does not show such an effect on 5637 cells cultured in serum-containing conditions, suggesting that Src activity is specifically required for viability of 5637 cells under serum-free conditions. On the other hand, AG99 shows a growth-inhibitory effect in both culture conditions, indicating that EGFR/kinase activity is required for both culture conditions. In 5637 cells, at least three types of Src family kinases are expressed; Src, Yes, and Fyn. Among them are Src and Yes that are activated in response to serum starvation [94]. Expression of kinase-negative Src also promotes apoptotic cell death in 5637 cells only under serum-free conditions (manuscript in preparation). Taken together, we conclude that Src activity is required for anti-apoptotic growth of 5637 cells under serum-free conditions. Further biochemical characterization of anti-apoptotic growth of serum-starved 5637 cells have demonstrated the following:

### Signal Transduction Leading to Anti-Apoptosis Involves MT1-MMP and HB-EGF

**Fig. (4):**
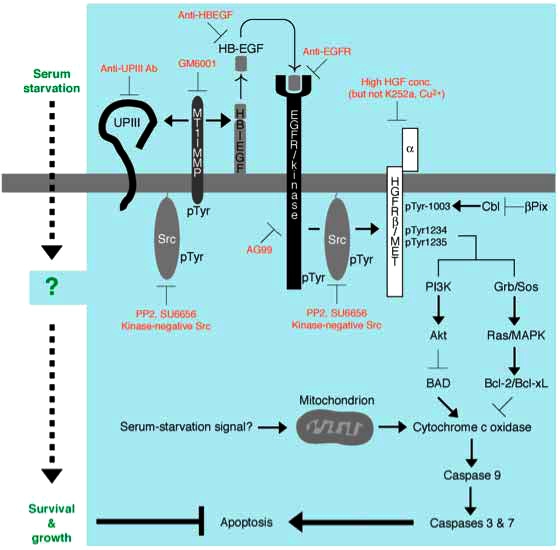
Anti-apoptotic mechanism of survival and growth of serum-starved 5637 human bladder carcinoma cells. Serum starvation signal may trigger both non-genomic (e.g. catalytic and/or physical protein interactions) and genomic responses (gene expression and/or protein synthesis) in 5637 cells. At early phase of signal transduction, activation of Src by unknown mechanism, activation of MT1-MMP by tyro-sine phosphorylation and/or increased protein expression, secretion of soluble HB-EGF into the culture medium, and proteolysis of uroplakin III are operating. Secreted HB-EGF binds to and activates EGFR/kinase. Src may also be activated under the control of EGFR. Src and EGFR phosphorylate β-subunit of HGFR/c-Met (p145) on tyrosine residues 1003, 1234, and 1235. These phosphotyrosine residues in β-subunit of HGFR/c-Met may be responsible for up-regulating anti-apoptotic ability of cells through PI 3-kinase-Akt pathway and/or Grb2-Sos-Ras-MAPK pathway: the former contributes to phosphorylation and inactivation of BAD, a pro-apoptotic component of Bcl-2 family, and the latter contributes to induction of Bcl-2 and Bcl-xL, both of which are anti-apoptotic components of Bcl-2 family. These components act positively or negatively on cytochrome c oxidase, which will be released from mitochondria upon mitochondria-mediated apoptosis in response to metabolically sensed death signals (i.e. serum starvation). Cytochrome c oxidase, if released, promotes sequential activation of caspase 9 and caspase 3/7, latter of which directly contributes to apoptotic cellular processes. Thus, serum-independent survival and growth of 5637 cells may involve suppression of pro-apoptotic pathway and/or activation of anti-apoptotic pathway

Among several ligands for EGFR/kinase [93] are heparin-binding EGF-like growth factor (HB-EGF [105,106]) that may serve as a main component to trigger EGFR/kinase activation in response to serum starvation of the cells. This is demonstrated by the facts that secretion of HB-EGF, but not EGF, into the culture medium is upregulated in serum-starved cells and that a specific antibody to HB-EGF, but not that to EGF, promotes apoptosis of serum-starved 5637 cells (manuscript in preparation). GM6001 [107], a potent inhibitor for matrix metalloproteinase (MMP) that is known to be an important regulator for secretion of HB-EGF and other EGFR/kinase ligands, also promotes apoptosis in serum-starved 5637 cells (manuscript in preparation). Among several kinds of MMPs is membrane type-1 MMP (MT1-MMP [108]) that may be involved in anti-apoptotic mechanism of serum-starved 5637 cells, because its protein expression is upregulated under serum-free culture conditions, and it becomes associated with Src and phosphorylated on tyrosine residues, which would act as an activating signal for MT1-MMP [109]. Thus, signal transduction pathway for anti-apoptotic growth of 5637 cells may involve expression of MT1-MMP by unknown mechanism, secretion of HB-EGF and its binding to EGFR/kinase, sequential activation of EGFR/kinase and Src, and phosphorylation of MT1-MMP (further activation of HB-EGF signal) and p145met (downstream signaling for anti-apoptosis, see below) (Fig. 4A). Importance of p145met in anti-apoptotic mechanism has been demonstrated as follows:

### p145^met^ is a Mediator of Anti-Apoptotic Function of Bladder Carcinoma Cells 

The fact that a potent inhibitor for p145^met^ K252a does not inhibit phosphorylation of p145^met^ and growth 5637 cells [94] suggests that catalytic activity of p145^met^ is not required for anti-apoptotic function of 5637 cells. However, another evidence that apoptosis in serum-starved 5637 cells is promoted by downregulation of p145^met^ by high concentrations of HGF [94], which causes endocytosis and breakdown of p145^met^ [110,111], indicates that the presence of tyrosine phosphorylated p145^met^ is required for anti-apoptotic mechanism. Mass spectrometric analysis of p145^met^ prepared from serum-starved cells demonstrates that tyrosine residues 1003, 1234, and 1235 are phosphorylated. Paradoxically, phosphorylation of tyrosine 1003 in p145^met^ has been shown to be important for the interaction with Cbl adaptor protein and subsequent ubiquitin-dependent degradation of the p145^met^ molecule [110]. Under this background, it will be interesting to examine expression of Cbl and β-Pix. β-Pix is an interacting protein for the PAK protein serine/threonine kinase, which counteracts the action of Cbl-dependent downregulation of receptor/kinases such as EGFR/kinase [112,113]. Thus, it is possible to think that in serum-starved 5637 cells, the expression level and/or functional level of β-Pix is more dominating than that of Cbl, thereby allows the phosphotyrosine 1003-containing p145^met^ molecule to be survived on the plasma membranes

### Possible Roles of Tyrosine Phosphorylation in p145^met^ : Speculations

On the other hand, phosphorylation of tyrosine residues 1234 and 1235 may be important more directly to anti-apoptotic mechanism. These phosphotyrosine residues are reportedly important for interaction with and activation of phosphatidylinositol 3-kinase (PI 3-kinase) [95, 114]. Activated PI 3-kinase is responsible for activation of serine/threonine kinase Akt that can phosphorylate and inactivate BAD [115], a proapoptotic protein that upregulates release of cytochrome c oxidase from mitochondria [116]. As cytochrome c oxidase is known to be a positive regulator of caspase 9, which in turn activates a release of active forms of downstream caspases 3 and 7, the signal cascade involving p145^met^-PI 3-kinase-Akt serves to act as anti-apoptotic mechanism. Phosphorylated p145^met^ may also recruit Grb2-Sos complex that can activate Ras, a small GTP-binding protein. Ras is well known to act upstream regulators of mitogen-activated protein serine/threonine kinase (MAPK) cascade [117]. Activated Ras-MAPK cascade has been shown to promote transcriptional activation of certain kind of genes such as Bcl-2 and Bcl-xL, both of which are anti-apoptosis components that act on the release of cytochrome c from mitochondria [118]. It is interesting to note that phosphorylation of BAD by the PI 3-kinase-Akt pathway is a relatively rapid, non-genomic process, whereas expression of Bcl-2 and Bcl-xL is a relatively slow, genomic process. Considering the fact that signal transduction of anti-apoptotic growth in serum-starved cells requires several hours for execution (e.g. ~8 hours for phosphorylation of p145^met^, see above), complex mode of actions involving both non-genomic and genomic processes may contribute to anti-apoptotic mechanisms in serum-starved 5637 cells. Future studies focusing on these pro-apoptotic and anti-apoptotic components, which would act downstream of p145^met^, will clarify which components are really functioning in this cancer cell system

## MEMBRANE MICRODOMAINS AND ANTI-APOPTOSIS

### Membrane Microdomains in Bladder Carcinoma Cells 

All proteins so far identified to be important for anti-apoptotic mechanism in 5637 cells have property to associate with plasma membranes (i.e. MT1-MMP, HB-EGF, EGFR/kinase, Src and p145^met^). Therefore, like in *Xenopus* egg system discussed above, we have an interest to analyze the structure of membrane microdomains of 5637 cells and its physiological importance in anti-apoptotic growth under serum-free conditions. Under this background, we have found that methyl-β-cyclodextrin inhibits tyrosine phosphorylation of EGFR/kinase, Src and p145^met^, and promotes apoptosis in serum-starved 5637 cells [119], manuscript in preparation). The results strongly suggest that cholesterol-dependent membrane microdomains play an important role in anti-apoptotic mechanism in serum-starved 5637 cells. As in the case of *Xenopus* eggs, membrane microdomains of 5637 cells could have been prepared by extraction of cells with Triton X-100-containing buffer followed by fractionation of the cell extract with discontinuous sucrose density gradient ultracentrifugation. The resulting Triton X-100-insoluble, low-density fractions are analyzed for the presence and function (e.g. phosphorylation) of signaling molecules, in comparison with those in non-microdomain fractions that are collected as detergent-soluble, high-density fractions. Experiments with use of subcellular fractions have demonstrated the following:

### Anti-Apoptosis Involves Cholesterol-Dependent Membrane Microdomains

Both Src and p145^met^ localize permanently to the membrane microdomains in 5637 cells, and undergo tyrosine phosphorylation in response to serum starvation. On the other hand, EGFR/kinase localizes to membrane microdomains and becomes tyrosine-phosphorylated only after serum starvation of cells. Interestingly, treatment of cells with methyl-β-cyclodextrin results in the disappearance of all of these proteins from the membrane microdomains (manuscript in preparation). This is not due to protein degradation but rather due to re-localization to the non-microdomain fractions. Regardless of the presence of these proteins, however, tyrosine phosphorylation is almost completely blocked in methyl-β-cyclodextrin-treated cells [119]. These results suggest that not only protein assembly in membrane microdomains but also tyrosine kinase signaling are distorted by the disruption of cholesterol-dependent membrane architecture. Inability of these membrane microdomain-associated molecules to undergo tyrosine phosphorylation may be because of the lack of functional access to MT1-MMP and/or HB-EGF, whose subcellular localization and sensitivity to methyl-β-cyclodextrin are currently unknown.

### Possible Role of Uroplakin III in Anti-Apoptosis

Analysis of membrane microdomains of 5637 cells has also demonstrated a new insight of anti-apoptotic growth of this cell line. By the use of a specific antibody against the extracellular domain of *Xenopus* UPIII (see above), we have found that a 45-kDa protein homologous to *Xenopus* UPIII is present in membrane microdomains of 5637 cells. Reportedly, human UPIII migrates at 45-50 kDa on SDS-polyacrylamide gel electrophoresis [70,72]. Treatment of the membrane microdomain fraction with *N*-glycosidase F results in a reduction of molecular size of the 45-kDa protein to about 30 kDa, which matches with the calculated size of the core protein of human UPIII. Therefore, we conclude that the 45-kDa protein is human UPIII (hereafter designated as p45UPIII). Functional relevance of p45UPIII in anti-apoptotic growth of 5637 cells has been suggested by the following observations: Like UPIII in *Xenopus* eggs, p45UPIII localizes predominantly to membrane microdomains in 5637 cells (unpublished results). Treatment of 5637 cells with methyl-β-cyclodextrin causes a dissociation of p45UPIII from membrane microdomains, indicating that as in case of EGFR/kinase, Src and p145^met^, the association of p45UPIII with membrane microdomains depends on its cholesterol-enriched nature. Although a cytoplasmic tyrosine residue, which is phosphorylated in *Xenopus* egg fertilization (tyrosine 249 in *Xenopus* UPIII [67]), is conserved in p45UPIII (tyrosine 266 in p45UPIII [120]), it is not phosphorylated in serum-starved 5637 cells. On the other hand, it is demonstrated that serum starvation promotes partial proteolysis of p45UPIII in a time course that is similar to tyrosine phosphorylation of p145^met^ (it is evident at ~8 h of serum starvation) (unpublished results). Pharmacological characterization of partial proteolysis of p45UPIII has demonstrated that the proteolytic reaction can be blocked effectively GM6001, a potent MMP inhibitor, and some other protease inhibitors such as leupeptin. Interestingly, however, a synthetic peptide, which contains a potential proteolytic site in *Xenopus* eggs (Gly-Arg-Arg sequence, see above [38]), does not inhibit the proteolysis of p45UPIII in serum-starved 5637 cells. Considering that the amino acid sequence covered by the synthetic peptide is completely conserved in *Xenopus* and human UPIII, it is plausible to think that proteolysis of p45UPIII occurs in a different site(s) of the molecule

### Proteolysis of Uroplakin III in Serum-Starved Bladder Carcinoma Cells

Another evidence that a specific antibody against the extracellular domain of UPIII can block effectively the proteolysis of p45UPIII strongly suggests that proteolysis of p45UPIII occurs, as in the case of *Xenopus* UPIII, in the extracellular part of the molecule. Importantly, GM6001 or antibody against the UPIII extracellular domain promotes apoptotic cell death in serum-starved 5637 cells (unpublished results). These results raise the possibility that proteolysis of p45UPIII is required for anti-apoptotic mechanism in serum-starved 5637 cells. Furthermore, inhibitors for Src family kinases (PP2 and SU6656) also shows an inhibitory effect on proteolysis of p45UPIII, suggesting that Src activity acts on upstream event of proteolysis; and paradoxically, GM6001 has an inhibitory effect on activation of Src and tyrosine phosphorylation of p145^met ^(unpublished results). Thus, it seems that coordinate and complex network involving proteolysis and tyrosine phosphorylation drives the anti-apoptotic mechanism in 5637 cells. Further approach on membrane microdomains to identify other signaling molecules (e.g. targets of proteolytic action, substrates of tyrosine kinases) as well as gene targeting approach by using knockdown technique (e.g. knockdown of MT1-MMP, p45UPIII etc.) will be useful to understand the molecular basis of sensing of serum-starved culture conditions, executing anti-apoptotic mechanism, and survival and growth of human cancer cells.
